# Sex‐specific changes in appetite and energy intake in response to chronic resistance training in rats

**DOI:** 10.14814/phy2.71030

**Published:** 2026-07-30

**Authors:** Josh C. Kuzminski, Jared R. Rollingson, Connor Bunnage, Jana Katoch, Marc R. Bomhof

**Affiliations:** ^1^ Department of Kinesiology and Physical Education University of Lethbridge Lethbridge Alberta Canada

**Keywords:** appetite‐regulating hormones, body weight, energy compensation, exercise, resistance training

## Abstract

Resistance training (RT) is widely prescribed for health, yet its effects on energy compensation and appetite regulation remain poorly defined, particularly across sexes. This study examined the effects of chronic RT on energy intake and appetite‐regulating hormones in rats. Animals (*n* = 40) were assigned to female control, female RT, male control, or male RT and completed a 12‐week ladder‐based RT protocol. Energy intake was assessed longitudinally, and hormonal responses were measured during a resistance training exercise test (RTET). RT reduced total energy intake (*p* = 0.022) without altering energy intake relative to body weight. Males exhibited reduced body weight and energy intake, whereas females did not, despite greater relative training volume. Postexercise energy intake was elevated in RT groups (*p* = 0.029), indicating delayed compensatory feeding. Acyl‐ghrelin increased following RTET (*p* < 0.001) and was higher in RT and females (*p* < 0.001). Leptin, insulin, GLP‐1, and PYY were unchanged. These findings demonstrate sexually dimorphic energy compensation during RT, associated with ghrelin secretion rather than tonic appetite hormones. RT appears to shift compensation toward episodic, ghrelin‐mediated pathways, with females exhibiting greater sensitivity. This identifies an endocrine framework for RT‐induced energy compensation and emphasizes the need for sex‐specific approaches to exercise prescription and energy balance regulation.

## 1 INTRODUCTION

Exercise is a cornerstone of health, with extensive evidence demonstrating reductions in all‐cause, cardiovascular, and cancer mortality (Rahmati et al., 2025). Consistent with this, the World Health Organization identifies physical inactivity as a leading global mortality risk factor (World Health Organization, 2010). Despite this, the exercise literature remains disproportionately focused on aerobic modalities, with comparatively limited investigation of anaerobic exercise, particularly resistance training (RT) (Grgic & Mikulic, 2017; Phillips et al., 2023; Rubio‐Valles & Ramos‐Jimenez, 2025; Zheng et al., 2022).

This imbalance persists despite growing evidence that RT confers substantial health benefits, including reduced risk of type 2 diabetes, cancer incidence and mortality, cardiovascular disease, and all‐cause mortality (Grøntved et al., 2014; Nascimento et al., 2021; Phillips et al., 2023; Qadir et al., 2021; Saeidifard et al., 2020; Shailendra et al., 2022), as well as unique effects on skeletal muscle mass, strength, and bone health (Armamento‐Villareal et al., 2020; Mende et al., 2022; Phillips et al., 2023; Zhang et al., 2022). Moreover, RT participation has increased substantially in the general population, particularly among women (Kraemer et al., 2025; Unger et al., 2025). Together, these trends highlight a critical need to address gaps in mechanistic understanding of RT‐related physiological adaptations.

One unresolved area is the paradox of energy compensation. Acute aerobic exercise is associated with transient suppression of energy intake and increased energy expenditure, yet chronic exercise often fails to produce sustained weight loss (Balaguera‐Cortes et al., 2011; Donnelly et al., 2014; Thomas et al., 2012, 2015). This discrepancy suggests compensatory adaptations in energy balance, with evidence indicating energy compensation may offset up to ~84% of exercise‐induced energy expenditure (Riou et al., 2015). Mechanistically, this chronic exercise‐induced compensation is thought to involve increased energy intake (Beaulieu et al., 2016; Khajehnasiri et al., 2023).

Postexercise appetite suppression is seen through changes in hormones, specifically decreased orexigenic acyl‐ghrelin, and increased anorexigenic glucagon‐like peptide‐1 (GLP‐1) and possibly peptide YY (PYY) (McCarthy et al., 2024). While these responses are relatively well characterized for aerobic exercise, substantially less is known about energy compensation in response to RT. Acute RT induces anorectic effects similar to aerobic exercise, though often of smaller magnitude (Dorling et al., 2018; Panissa et al., 2024). However, limited evidence suggests that chronic RT may reduce energy intake overall, contrasting with patterns observed in aerobic exercise (Panissa et al., 2024). Given that RT elicits distinct endocrine and metabolic responses compared with aerobic exercise (Balaguera‐Cortes et al., 2011; Dorling et al., 2018), it is plausible that energy compensation mechanisms differ between exercise modalities.

Sex represents an additional, yet underexplored, modifier of energy compensation. Evidence from aerobic exercise studies suggests that females may exhibit greater compensatory responses to negative energy balance, particularly through increased energy intake (Foright et al., 2020). However, findings are inconsistent, with some studies reporting sex‐specific effects in females, others in males, and some showing no differences (Stubbs, Sepp, Hughes, Johnstone, Horgan, et al., 2002; Stubbs, Sepp, Hughes, Johnstone, King, et al., 2002; Whybrow et al., 2008). Notably, this literature is almost exclusively derived from aerobic exercise paradigms. This represents a significant limitation, as well‐established sex differences exist in skeletal muscle adaptation and physiological responses to RT (Landen et al., 2023) suggesting that compensatory mechanisms may also differ by sex in this context.

Collectively, there is a clear lack of research examining chronic RT, particularly in females, and an absence of studies investigating sex‐specific differences in appetite regulation and energy compensation during chronic RT. Therefore, the primary objective of this study was to examine the effects of chronic RT on appetite‐regulating hormones and energy intake in male and female Sprague Dawley rats. We hypothesized that RT would elicit acute anorectic responses in both sexes, but that compensatory increases in energy intake would occur over time, with a greater magnitude of energy compensation in females relative to males.

## 2 MATERIALS AND METHODS

### 2.1 Animals and experimental protocol

All procedures conformed to the guidelines of the Canadian Council on Animal Care and were approved by the University of Lethbridge Animal Welfare Committee (Protocol #2503). All efforts were made to minimize animal suffering and reduce the number of animals used. Forty 10‐week‐old Sprague Dawley rats (*n* = 40; 20 male, 20 female) were obtained from Charles River Laboratories (St. Constant, PQ, Canada). Animals were individually housed in Techniplast double‐decker cages with solid floors (Toronto, ON, Canada) with environmental enrichment including shredded paper towels, crinkle paper bedding, and PVC tubes. Rats were maintained in temperature‐controlled rooms (20–22°C) with controlled humidity and a 12‐h light–dark cycle (07:00–19:00). Cages were cleaned weekly and animals were monitored daily to ensure absence of distress. Rats underwent a 7‐day acclimatization period with ad libitum access to water and standardized AIN‐93 M purified diet (#110700, Dyets Inc., Bethlehem, PA, USA; 3.6 kcal/g) and familiarization to ladder‐based RT. Animals were randomly assigned using a computer‐generated randomization sequence into one of four groups (*n* = 10/group): female control (female C), female RT, male control (male C), and male RT.

### 2.2 Energy intake and body weight

All rats were provided ad libitum access to a standardized AIN‐93 M purified diet (#110700, Dyets Inc., Bethlehem, PA, USA; 3.6 kcal/g). Food intake was measured as the difference between food provided and food remaining, expressed as grams of AIN‐93 M purified diet consumed. Intake was measured 5 days per week with weekend intake recorded as a single combined measurement during weeks 1–11. Energy intake was calculated by multiplying food intake by the manufacturer‐provided energy density of the diet (3.6 kcal/g). During week 12, intake was measured twice daily at the beginning of the light and dark cycles. Training‐day and rest‐day energy intake were calculated for RT groups. Body weight was measured weekly and prior to all testing procedures.

### 2.3 RT protocol

A ladder‐based RT model was used (1.1 m × 0.18 m; 2‐cm grid steps; 80° incline) with a resting chamber at the top (Hornberger Jr. & Farrar, 2004). Loads were attached to the base of the tail using adhesive tape and a tether system. During familiarization, rats underwent ladder training with a load equivalent to 5% of body weight. A gentle tactile stimulus was applied to initiate climbing when required. A repetition was considered successful when the rat climbed voluntarily without assistance. Training continued until three consecutive successful repetitions were achieved. Following a 48‐h rest period, maximal load (ML) was determined. Rats climbed with an initial load of 75% body weight, with 30 g increments added after each successful climb until failure occurred on two consecutive attempts. The highest successfully completed load was recorded as ML. RT groups completed a progressive protocol for 11 weeks (3 sessions/week during weeks 1–2; 4 sessions/week during weeks 3–11) (Krause Neto et al., 2022; Luciano et al., 2017; Padilha et al., 2019; Tibana et al., 2017). Each session consisted of: 50% ML (1 climb), 75% ML (2 climbs), 90% ML (2 climbs), 100% ML (2 climbs), and 100% ML + 30 g (0–2 climbs), with 90‐s rest between climbs. The final two climbs of each session were used to assess whether the animal could exceed its current ML. These attempts were performed only if all preceding climbs were completed successfully. For the first attempt, 30 g was added to the animal's current ML. If this climb was completed successfully, the new load was recorded as the updated ML and a second attempt was performed with an additional 30 g. If the first attempt was unsuccessful, the animal was given one additional attempt at the same load. Training volume (load × successful climbs) was recorded per session and per week. Control animals were transported and cages were handled equivalently to match experimental stress.

### 2.4 RTET and blood collection

During week 12, a resistance training exercise test (RTET) was conducted following 48 h of rest and a 12‐h overnight fast. The RTET protocol matched the maximal load test for controls, while resistance‐trained animals began at 50% ML to account for training adaptations. Blood was collected via lateral tail vein sampling. Rats were placed on a heating pad to facilitate vasodilation. The sampling site was disinfected with chlorhexidine. A 21‐gage butterfly needle was modified to have the tube behind the wings removed and then used for collection. Baseline samples (100 μL) were collected prior to RTET, followed by immediate postexercise and subsequent samples at 30, 60, and 120 min. Blood was collected into chilled EDTA‐coated microtainer tubes containing dipeptidyl peptidase IV inhibitor (10 μL/mL; #DPP4‐M, MilliporeSigma Corp. Burlington, MA, USA), sigma protease inhibitor (1 mg/mL; #S8820, SigmaFast, MilliporeSigma Corp. Burlington, MA, USA), and Pefabloc (1 mg/mL; #76307, MilliporeSigma Corp. Burlington, MA, USA) and immediately placed on ice. Blood glucose and lactate were measured using a OneTouch Glucose Meter (Lifescan Inc., Malvern, PA, USA), a Lactate Plus Meter (NOVA Biomedical, Mississauga, ON, Canada), and Lactate Plus Meter Test Strips (#40813, NOVA Biomedical, Mississauga, ON, Canada). Samples were centrifuged at 1200 × g for 10 min at 4°C, and plasma was stored at −80°C.

### 2.5 Biochemical analysis

Plasma concentrations of acyl‐ghrelin, GLP‐1, PYY, insulin, and leptin were quantified simultaneously from each biological sample using U‐PLEX metabolic group 1 (rat) multiplex assays (#K153ACM‐21, Meso Scale Diagnostics, Rockville, MD, USA). All biological samples were assayed in singlicate, with standards and quality controls prepared according to the manufacturer's instructions. Samples were analyzed on 96‐well plates with balanced group representation per plate, following manufacturer protocols. Detection was performed using a MESO QuickPlex SQ 60 instrument and associated MethodicalMind software.

### 2.6 Estrous cycle assessment

Female rats were staged via vaginal smear during week 11 and on the day of RTET (Raimondi et al., 2023). Samples were collected at the beginning of the light phase using saline‐moistened cotton swabs inserted ~1 cm into the vaginal canal. Samples were transferred to slides, dried, stained with Dip Quick Stain (#J0322 Series, Jorgensen Laboratories Inc., Loveland, CO, USA), and imaged at 10× magnification. Estrous stage was classified using EstrousNet (Wolcott et al., 2022).

### 2.7 Tissue and portal blood collection

Following a 12‐h fast, rats were anesthetized with isoflurane (5% induction, 2% maintenance). Portal vein blood (3 mL) was collected using a 21‐gage butterfly needle and syringe. Following portal vein blood collection, the heart was excised to induce terminal exsanguination. Fat depots (retroperitoneal, perirenal, epididymal/parametrial, inguinal) were excised and weighed. Relative fat mass was calculated as total fat mass divided by final body weight. Skeletal muscles (FHL, tibialis posterior, gastrocnemius, plantaris, soleus, tibialis anterior, FDL, biceps brachii) were excised and weighed.

### 2.8 Statistical analysis

Data were analyzed using IBM SPSS Statistics (version 31.0; IBM Corp., Armonk, NY, USA). Normality was assessed using standardized skewness and kurtosis (*z*‐scores), with values exceeding ±3.29 considered indicative of nonnormality, and appropriate transformations applied where necessary. The primary outcomes were circulating appetite‐regulating hormones and energy intake. Secondary outcomes included body weight, tissue mass, and performance variables. For repeated measures outcomes, two‐way repeated‐measures ANOVAs were conducted with time as the within‐subject factor and sex (male, female) and/or condition (control, RT) as between‐subject factors. When Mauchly's test indicated a violation of sphericity, Greenhouse–Geisser corrections were applied. For single time‐point and area under the curve (AUC) outcomes, two‐way ANOVA was used with sex and condition as between‐subject factors. Significant group/condition by sex interactions were followed by one‐way ANOVA within relevant factors. Post hoc comparisons were performed using Tukey's test when homogeneity of variance was met and Dunnett's T3 when violated. AUC was calculated using the trapezoidal method. Statistical significance was set at *p* < 0.05. Data are presented as mean ± standard deviation (SD). An a priori power analysis was conducted in G*Power (version 3.1.9.7) using an F‐test for repeated‐measures ANOVA assessing the within–between interaction (4 groups, 5 repeated measurements, α = 0.05, desired power of 0.80, repeated‐measures correlation of 0.50, and nonsphericity correction ε set to 0.75). Effect size estimates were informed by prior work (Durocher et al., 2025) examining postprandial hormone responses in exercising rodent models, where significant group‐by‐time interactions were observed, corresponding to moderate effect sizes. Given differences in study design and to provide a conservative estimate, a medium effect size (f = 0.25) was selected for the primary power analysis.

## 3 RESULTS

### 3.1 Body weight

**FIGURE 1:**
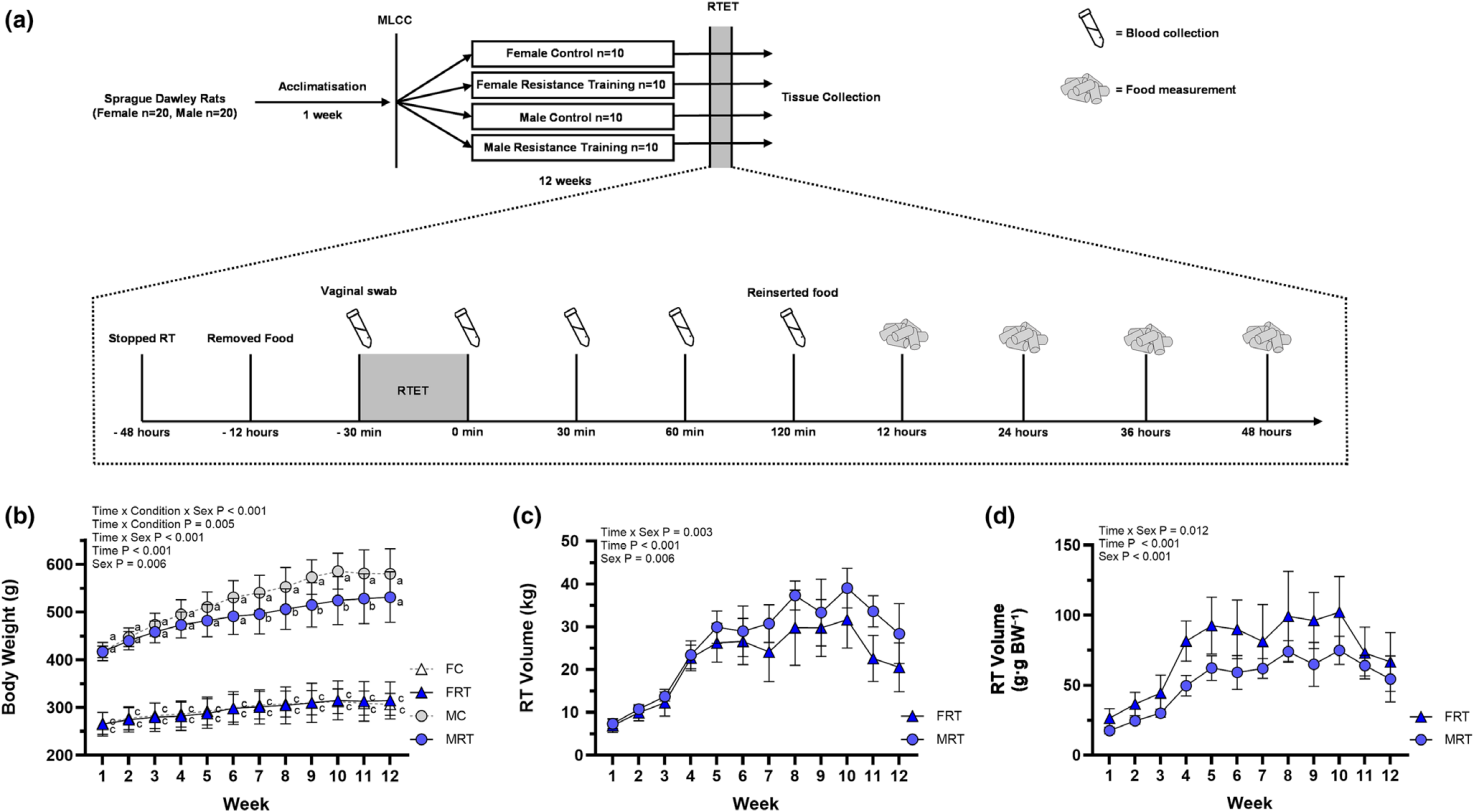
Schematic of study protocol (a), Weekly body weight (b), Exercise volume (c), and Exercise volume relative to body weight (d). Values are means ± SD. Two‐way repeated measures ANOVAs were used. Significant interactions of (b) were followed by one‐way ANOVAs within relevant factors. Post hoc comparisons were performed using Tukey's test when homogeneity of variance was met and Dunnett's T3 when violated. *n* = 10 for FC, FRT, MC, and MRT. When a Condition × Sex effect was observed, ^abc^superscripts indicate significant group differences, labeled means at a time without a common letter differ, *p* < 0.05. Volume was calculated as the sum of repetitions × weight for each week. FC, female control; FRT, female resistance training; MC, male control; MLCC, max load carrying capacity; MRT, male resistance training; RTET, resistance training exercise test.

Baseline body weights were similar between groups (*p* = 0.963). During the 12‐week intervention, all groups increased body weight over time (time, *p* < 0.001), with significant time × sex (*p* < 0.001), time × condition (*p* = 0.005), and time × condition × sex interactions (*p* < 0.001). These effects reflected greater body weight gain in males than females and a condition‐specific effect of RT in males. Specifically, male C had higher body weight than male RT during weeks 7–11, whereas body weight did not differ between female C and female RT. Across the intervention period, males remained heavier than females (sex, *p* < 0.001) (Figure 1b).

### 3.2 Training volume

Training volume increased over time (time, *p* < 0.001), with males showing a greater increase in absolute training volume than females (time × sex, *p* = 0.003). Overall absolute training volume was also higher in male RT than female RT (sex, *p* = 0.006). When training volume was adjusted for body weight, the pattern differed: body weight‐adjusted volume increased over time (time, *p* < 0.001), but females showed greater increases and higher overall body weight‐adjusted training volume than males (time × sex, *p* = 0.012; sex, *p* < 0.001) (Figure 1[c,d]).

### 3.3 Energy intake over 12 weeks

**FIGURE 2:**
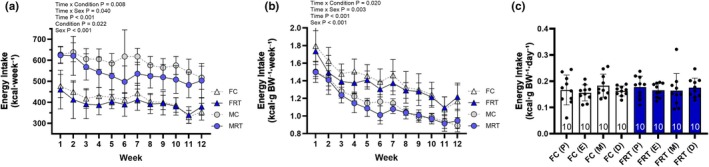
Weekly EI, expressed as kcal/week from weeks 1 to 12 (a), relative weekly EI kcal/week per g of body weight from weeks 1 to 12 (b), and EI by female estrous cycle stage during week 11 (c). Values are means ± SD. Two‐way repeated‐measures ANOVAs were used for (a and b), and a two‐way ANOVA was used for (c). *n* = 10 for FC, FRT, MC, and MRT. D, diestrus; E, estrus; FC, female control; FRT, female resistance training; M, metestrus; MC, male control; MRT, male resistance training; P, proestrus; RTET, resistance training exercise test.

Total energy intake decreased over time across the 12‐week intervention (time, *p* < 0.001), with greater reductions over time in RT animals and males (time × condition, *p* = 0.008; time × sex, *p* = 0.040). Total energy intake was also lower overall in RT compared with control animals (condition, *p* = 0.022) and in females compared with males (sex, *p* < 0.001). However, when energy intake was normalized to body weight, relative energy intake also decreased over time (time, *p* < 0.001), with greater reductions over time in RT animals and females (time × condition, *p* = 0.020; time × sex, *p* = 0.003). Overall relative energy intake was lower in males than females (sex, *p* < 0.001) (Figure 2).

### 3.4 Energy intake and estrous cycle

No effects were observed for total or body weight‐adjusted intake across estrous cycle stages (Figure 2c).

### 3.5 Energy intake on rest and training days

**FIGURE 3:**
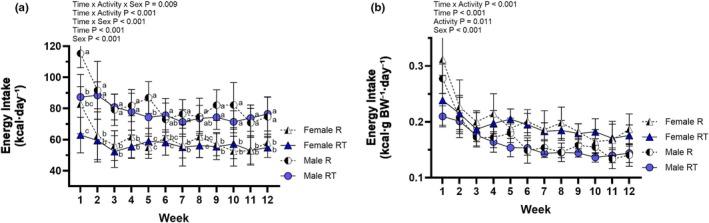
Total EI kcal/day (a) and Relative EI kcal per g of body weight (b) on training and rest days over 12 weeks in RT groups. Values are means ± SD. Two‐way repeated‐measures ANOVAs were used. Significant interactions of (a) were followed by one‐way ANOVAs within relevant factors. Post hoc comparisons were performed using Tukey's test when homogeneity of variance was met and Dunnett's T3 when violated. *n* = 10 for, FRT R, FRT RT, MRT R, and MRT RT. When an Activity × Sex effect was observed, ^abc^superscripts indicate significant group differences, labeled means at a time without a common letter differ, *p* < 0.05. FRT, female resistance training; MRT, male resistance training; R, rest days; RT, resistance training days.

Total energy intake decreased over time (time, *p* < 0.001), with significant time × activity (*p* < 0.001) and time × sex interactions (*p* < 0.001). These effects reflected differences in intake between training and rest days that varied across the intervention, as well as higher total energy intake in males than females (sex, *p* < 0.001). Follow‐up analyses showed that total energy intake was higher on rest days than training days during week 1 in both sexes and during week 5 in males (*p* ≤ 0.05). Males also consumed more total energy than females across most training and rest day time points (*p* ≤ 0.05) (Figure 3a). Body weight‐adjusted energy intake also decreased over time (time, *p* < 0.001), with a significant time × activity interaction (*p* < 0.001), indicating that the difference between training and rest days varied across weeks. Overall, body weight‐adjusted energy intake was higher on rest days than training days (activity, *p* = 0.011) and higher in females than males (sex, *p* < 0.001) (Figure 3b).

### 3.6 Post‐RTET 48 h energy intake

**FIGURE 4:**
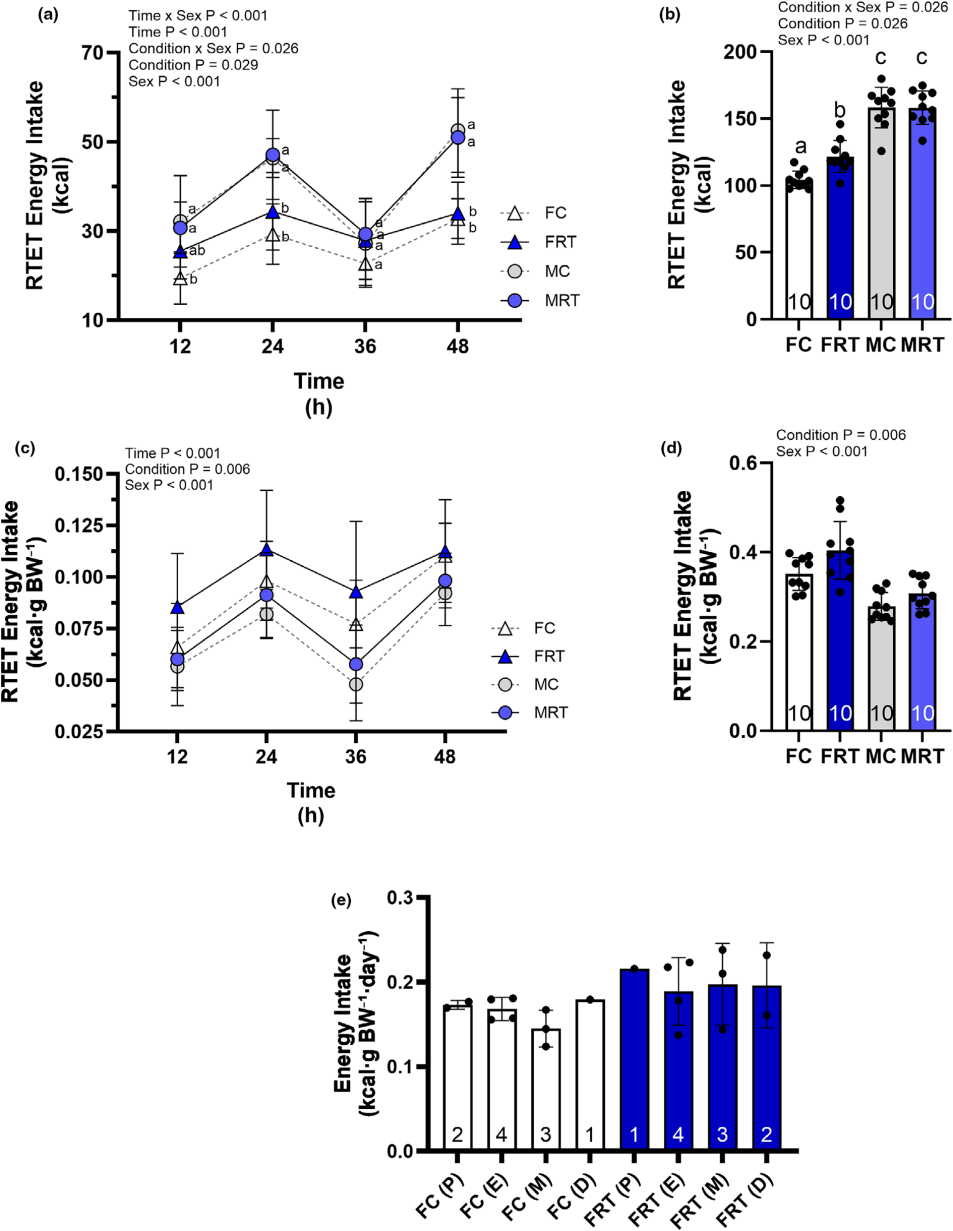
48‐h EI over time post‐RTET kcal (a), Total 48‐h EI post‐RTET kcal (b), Relative 48‐h EI over time post‐RTET kcal per g of body weight (c), Total relative 48‐h EI post‐RTET kcal per g of body weight (d), Relative 24‐h EI post‐RTET kcal per g of body weight by female estrous stage and condition (e). Values are means ± SD. Two‐way repeated‐measures ANOVAs were used for (a and c), and two‐way ANOVAs were used for (b and d). Significant interactions of (a and b) were followed by one‐way ANOVAs within relevant factors. Post hoc comparisons were performed using Tukey's test when homogeneity of variance was met and Dunnett's T3 when violated. *n* = 10 for FC, FRT, MC, and MRT. When a Condition × Sex effect was observed, ^abc^superscripts indicate significant group differences, labeled means at a time without a common letter differ, *p* < 0.05. D, diestrus; E, estrus; FC, female control; FRT, female resistance training; M, metestrus; MC, male control; MRT, male resistance training; P, proestrus; RTET, resistance training exercise test.

Post‐RTET energy intake varied over time (time, *p* < 0.001), with intake peaking during dark‐cycle measurement periods and greater temporal changes in males than females (time × sex, *p* < 0.001). Overall post‐RTET energy intake was higher in RT than control animals (condition, *p* = 0.029) and higher in males than females (sex, *p* < 0.001), with a significant condition × sex interaction (*p* = 0.026). These effects reflected lower energy intake in female C compared with both male groups at 12, 24, and 48 h, and lower energy intake in female RT compared with both male groups at 24 and 48 h (all *p* ≤ 0.05). Summed 48‐h energy intake showed the same pattern, with significant effects of condition (*p* = 0.026), sex (*p* < 0.001), and condition × sex (*p* = 0.026). Specifically, female C had lower 48‐h energy intake than all other groups, while female RT had lower 48‐h energy intake than both male groups (all *p* ≤ 0.05). Body weight‐adjusted post‐RTET energy intake also varied over time (time, *p* < 0.001), with higher relative intake in RT than control animals (condition, *p* = 0.006) and higher relative intake in females than males (sex, *p* < 0.001). Summed body weight‐adjusted 48‐h energy intake showed the same condition and sex effects (condition, *p* = 0.006; sex, *p* < 0.001) (Figure 4).

### 3.7 Estrous cycle energy intake post‐RTET

Estrous cycle‐stage post‐RTET energy intake data are presented descriptively only because the number of animals within each stage was insufficient for adequately powered group comparisons (Figure 4e).

### 3.8 Biomarkers surrounding RTET

**FIGURE 5:**
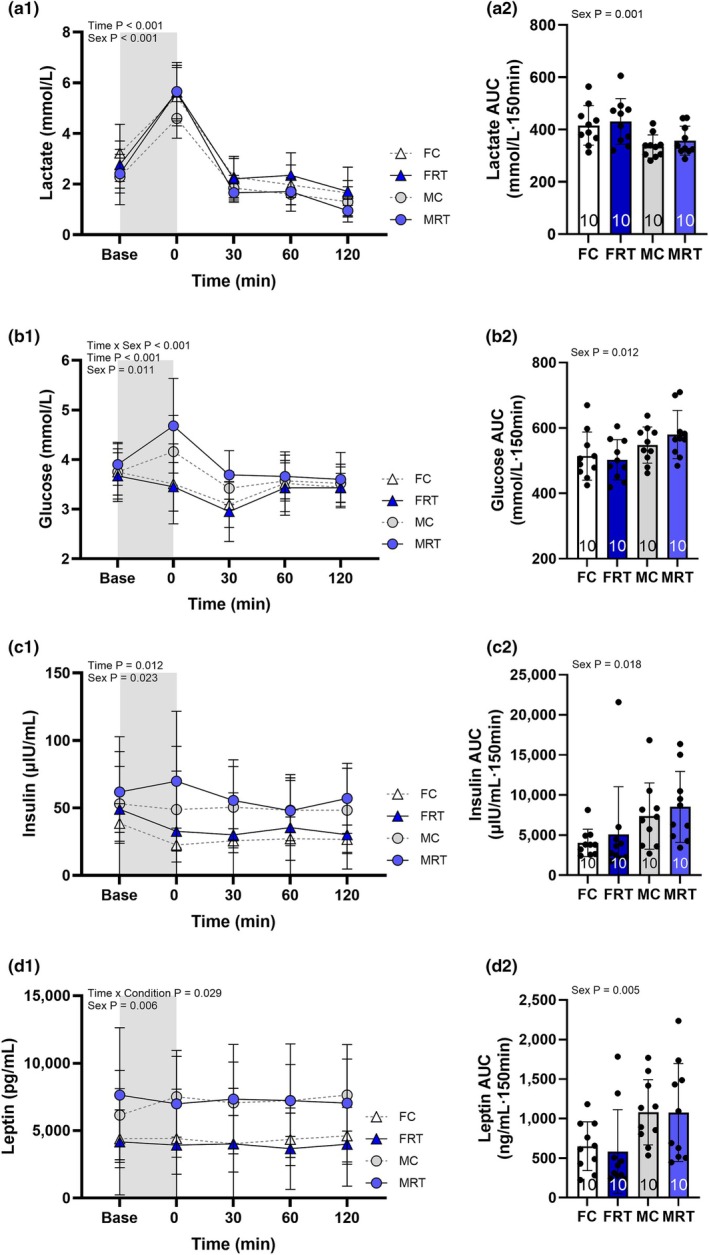
Glucose and long‐term appetite‐regulating hormones. Concentrations (1) and area under the curve (AUC) (2) for Lactate (a), Glucose (b), Insulin (c), and Leptin (d) during a 120‐min resistance training exercise test. Values are means ± SD. Two‐way repeated‐measures ANOVAs were used for (1), and two‐way ANOVAs were used for (2). *n* = 10 for FC, FRT, MC, and MRT. Gray shading shows the time of resistance training exercise test. FC, female control; FRT, female resistance training; MC, male control; MRT, male resistance training.

**FIGURE 6:**
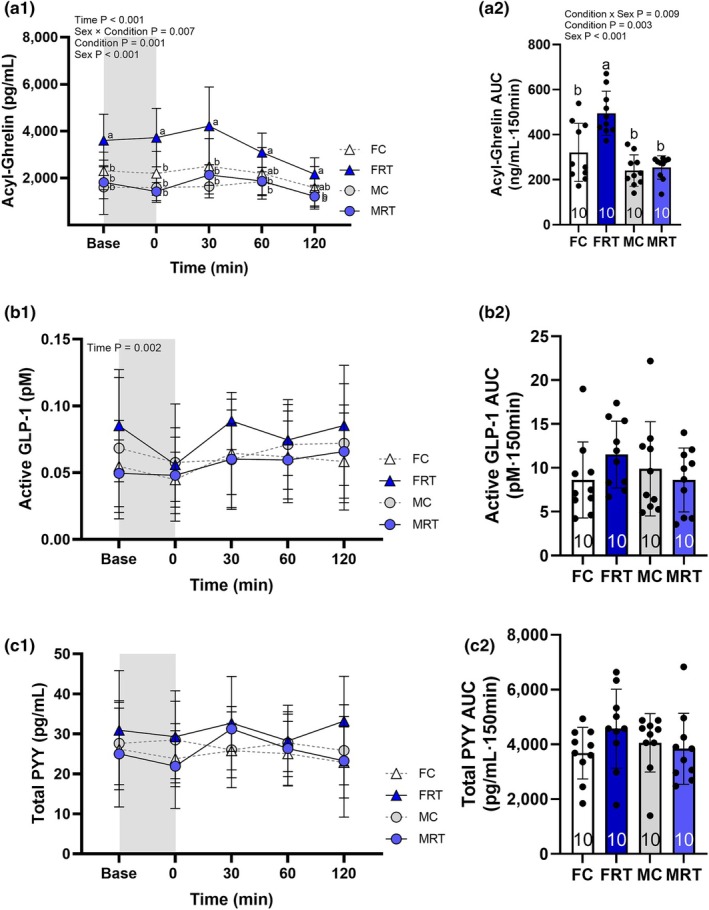
Episodic appetite‐regulating hormones. Concentrations (1) and area under the curve (AUC) (2) for Acyl‐Ghrelin (a), Active GLP‐1 (b), and Total PYY (c) during a 120‐min resistance training exercise test. Values are means ± SD. Two‐way repeated‐measures ANOVAs were used for (1), and two‐way ANOVAs were used for (2). Significant interactions of (a1 and a2) were followed by one‐way ANOVA within relevant factors. Post hoc comparisons were performed using Tukey's test when homogeneity of variance was met and Dunnett's T3 when violated. *n* = 10 for FC, FRT, MC, and MRT. When a Condition × Sex effect was observed, ^ab^superscripts indicate significant group differences, labeled means at a time without a common letter differ, *p* < 0.05. Gray shading shows the time of resistance training exercise test. FC, female control; FRT, female resistance training; GLP‐1, glucagon‐like peptide 1; MC, male control; MRT, male resistance training; PYY, peptide YY.

Lactate increased following RTET (time, *p* < 0.001) and was higher in females than males across the sampling period (sex, *p* < 0.001). Lactate AUC showed the same sex effect, with females having higher overall lactate than males (sex, *p* < 0.001) (Figure 5). Glucose also varied over time (time, *p* < 0.001), with a significant time × sex interaction (*p* < 0.001). This reflected divergent post‐RTET responses, with glucose increasing in males and decreasing in females. Overall glucose concentrations were higher in males than females (sex, *p* < 0.001), which was confirmed by AUC analysis (sex, *p* < 0.001). Insulin varied over time (time, *p* = 0.012) and was higher in males than females across the sampling period (sex, *p* = 0.023), with AUC analysis showing the same sex effect (sex, *p* = 0.018). Leptin showed a significant time × condition interaction (*p* = 0.029), reflecting a post‐RTET decrease in RT animals that was not observed in controls. Leptin concentrations were also higher in males than females (sex, *p* = 0.006), and this sex effect was confirmed by AUC analysis (sex, *p* = 0.005). Acyl‐ghrelin increased post‐RTET (time, *p* < 0.001), peaking at 30 min postexercise. Acyl‐ghrelin was higher in RT than control animals (condition, *p* < 0.001) and higher in females than males (sex, *p* < 0.001), with a significant condition × sex interaction (*p* = 0.007). Follow‐up analyses showed that female RT had higher acyl‐ghrelin than both male groups at all time points and higher acyl‐ghrelin than female C at baseline, 0 min, and 30 min post‐RTET (all *p* ≤ 0.05). Acyl‐ghrelin AUC showed the same pattern, with significant condition × sex (*p* = 0.009), condition (*p* = 0.003), and sex effects (*p* < 0.001) (Figure 6). Active GLP‐1 varied over time (time, *p* = 0.002), decreasing immediately post‐RTET before recovering.

### 3.9 Portal hormones

**FIGURE 7:**
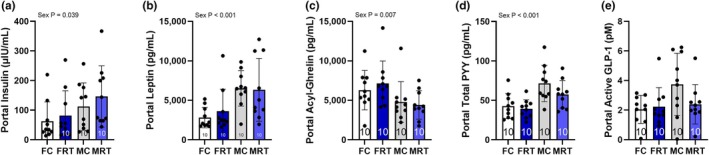
Fasting portal blood concentrations of appetite‐regulating hormones after 12 weeks. Portal Insulin (a), Leptin (b), Acyl‐Ghrelin (c), Total PYY (d), and GLP‐1 (e). Values are means ± SD. Two‐way ANOVAs were used. *n* = 10 for FC, FRT, MC, and MRT. FC, female control; FRT, female resistance training; GLP‐1, glucagon‐like peptide 1; MC, male control; MRT, male resistance training; PYY, peptide YY.

Fasting portal hormone concentrations differed by sex. Males had higher insulin (sex, *p* = 0.039), leptin (sex, *p* < 0.001), and total PYY (sex, *p* < 0.001) than females, whereas females had higher acyl‐ghrelin than males (sex, *p* = 0.007) (Figure 7).

### 3.10 Body measurements

Males had greater raw fat mass overall and in all depots except the perirenal depot (sex, *p* < 0.001). When expressed relative to body weight, males had greater inguinal (sex, *p* < 0.001) and retroperitoneal fat mass (sex, *p* = 0.039), whereas females had greater relative perirenal fat mass (sex, *p* = 0.037) (Table 1). Raw muscle weights were also higher in males across all measured muscles, including total collected muscle mass (sex, *p* < 0.001). Tibialis anterior weight showed a sex × condition interaction; however, follow‐up comparisons did not identify significant pairwise group differences beyond the main sex effect. In contrast to raw muscle mass, body weight‐normalized muscle mass was greater in females for the gastrocnemius (sex, *p* = 0.016), tibialis anterior (sex, *p* < 0.001), FDL (sex, *p* < 0.001), biceps brachii (sex, *p* < 0.001), and total collected muscle mass (sex, *p* < 0.001) (Table 1). RT animals had greater body weight‐normalized FDL (condition, *p* = 0.006), biceps brachii (condition, *p* = 0.044), and total collected muscle mass (condition, *p* = 0.041) than controls (Table 1).

**TABLE 1:** Body weights and relative tissue weights.

Variable mean ± SD	Female control (*N* = 10)	Female RT (N = 10)	Male control (N = 10)	Male RT (N = 10)	Condition	Sex	Condition × Sex
Initial Body Weight (g)	257.2 ± 20.83	256.7 ± 21.85	406.9 ± 17.72	406.8 ± 14.16	*p* = 0.960	***p* < 0.001**	*p* = 0.973
Final Body Weight (g)	294.61 ± 28.27^b^	308.23 ± 37.74^b^	566.88 ± 48.29^a^	524.21 ± 52.11^a^	*p* = 0.283	***p* < 0.001**	***p* = 0.042**
Inguinal Fat (g/kg BW)	16.52 ± 7.69	16.22 ± 6.30	28.74 ± 9.74	24.65 ± 9.29	*p* = 0.421	***p* < 0.001**	*p* = 0.487
Epididymal/Parametrial Fat (g/kg BW)	22.69 ± 7.51	22.48 ± 9.19	20.02 ± 4.48	18.64 ± 4.74	*p* = 0.707	*p* = 0.130	*p* = 0.782
Perirenal Fat (g/kg BW)	10.40 ± 5.19	8.47 ± 2.72	7.75 ± 5.15	5.68 ± 1.60	*p* = 0.121	***p* = 0.037**	*p* = 0.956
Retroperitoneal (g/kg BW)	16.74 ± 5.50	16.04 ± 5.91	21.29 ± 6.67	19.49 ± 5.76	*p* = 0.509	***p* = 0.039**	*p* = 0.771
Total Fat (g/kg BW)	66.35 ± 20.19	63.21 ± 21.76	77.81 ± 17.02	68.46 ± 19.41	*p* = 0.324	*p* = 0.189	*p* = 0.623
FHL and Tibialis Posterior (g/kg BW)	1.06 ± 0.12	1.06 ± 0.18	0.95 ± 0.12	1.05 ± 0.12	*p* = 0.232	*p* = 0.192	*p* = 0.255
Gastrocnemius (g/kg BW)	5.42 ± 0.32	5.64 ± 0.65	5.01 ± 0.47	5.29 ± 0.38	*p* = 0.103	***p* = 0.016**	*p* = 0.825
Plantaris (g/kg BW)	1.06 ± 0.10	1.07 ± 0.11	0.98 ± 0.15	1.03 ± 0.20	*p* = 0.528	*p* = 0.211	*p* = 0.672
Soleus (g/kg BW)	0.49 ± 0.04	0.47 ± 0.09	0.42 ± 0.08	0.47 ± 0.05	*p* = 0.485	*p* = 0.192	*p* = 0.103
Tibialis Anterior (g/kg BW)	1.92 ± 0.14	2.01 ± 0.29	1.71 ± 0.14	1.74 ± 0.20	*p* = 0.368	***p* < 0.001**	*p* = 0.674
FDL (g/kg BW)	2.27 ± 0.22	2.45 ± 0.29	1.96 ± 0.12	2.19 ± 0.21	***p* = 0.006**	***p* < 0.001**	*p* = 0.719
Biceps Brachii (g/kg BW)	1.33 ± 0.13	1.44 ± 0.22	1.14 ± 0.18	1.25 ± 0.16	***p* = 0.044**	***p* = 0.001**	*p* = 0.958
Collected Muscle (g/kg BW)	13.54 ± 0.90	14.13 ± 1.45	12.16 ± 0.89	13.02 ± 0.97	***p* = 0.041**	***p* < 0.001**	*p* = 0.696

*Note*: Values are means ± SD. *n* = 10 for FC, FRT, MC, and MRT. ^ab^Superscripts indicate significant group differences, labeled means at a variable without a common letter differ, *p* < 0.05. Significant values are in bold.

Abbreviations: FDL, flexor digitorum longus; FHL, flexor hallucis longus; RT, resistance training.

## 4 DISCUSSION

To our knowledge, this is the first study to examine energy compensation and appetite hormone responses to chronic RT under controlled conditions across sexes. We observed that RT, relative to sedentary controls, led to alterations in energy intake characterized by reductions in total intake without changes when normalized to body weight. Importantly, females demonstrated greater energy compensation in response to RT compared to males, despite completing a higher relative training volume. This compensatory response was associated with elevated postexercise acyl‐ghrelin in females, suggesting a potential role for ghrelin in mediating sex‐specific adaptations in energy balance. Notably, and in contrast to findings commonly reported following aerobic exercise (McCarthy et al., 2024), RT did not produce changes in other key appetite‐regulating hormones, including leptin, insulin, GLP‐1, or PYY. Together, these findings indicate that RT elicits distinct, sex‐dependent adaptations in energy balance.

This study utilized a ladder climbing model to investigate energy compensation in response to RT. The validity of this model is supported by prior rodent work, including systematic evidence (Lourenço et al., 2020), showing that ladder‐based protocols induce skeletal muscle hypertrophy. In the present study, RT animals exhibited greater relative muscle weights compared to sedentary controls, and training volume progressively increased over the first 10 weeks, indicating an effective progressive overload stimulus. The modest decline in volume during weeks 11–12 likely reflects increased testing burden and additional rest periods rather than failure of the training paradigm. Females completed more relative training volume, which is consistent with previous aerobic research showing females complete more physical activity than males (Mathis et al., 2024).

RT reduced total energy intake compared to controls over 12 weeks, while relative energy intake remained unchanged, indicating that reductions in intake occurred alongside changes in body mass rather than reflecting suppression relative to energetic demand. The lower body weight observed in male RT compared with male C from week 7 onward suggests that RT attenuated body weight gain in males without producing sustained reductions in body weight‐adjusted energy intake. Although relative energy intake did not differ overall across the 12‐week intervention, RT groups showed faster early decreases that converged toward control groups during the latter half of training. This pattern may indicate that exercise initially reduced energy intake, followed by delayed energy compensation through intake in an attempt to restore energy balance. This delay in energy compensation has been shown in previous research in male rats who initially reduce intake and body weight before compensatory increases restore energy balance (Durocher et al., 2025) and fits with the current model of exercise energy compensation, which states that energy compensation increases with intervention duration (Riou et al., 2015). Interestingly, RT groups exhibited higher relative and total energy intake over the 48‐h following the resistance exercise challenge test conducted during the final week of the study. To our knowledge, this is the first study to assess an acute appetite response in RT versus untrained animals. The elevated energy intake response in the postexercise period and tendency for increased intake on rest days compared to training days suggests that compensatory feeding may be distributed across recovery periods rather than occurring immediately postexercise. Human evidence examining chronic RT effects on energy intake remains limited and heterogeneous. Our findings contrast Panissa et al. (2024) who reported reductions in energy intake with RT, although these findings were largely confined to populations with overweight or obesity. Recent RT‐only studies in healthy females and males reported increased orexigenic drive after 6–8 weeks of RT without corresponding increases in total energy intake (Barros et al., 2024; Cardoso et al., 2024), suggesting that appetite‐related signals may change without producing a sustained increase in chronic energy intake. In contrast, Bales et al. (2012) reported no increase in absolute energy intake or energy intake relative to body mass after 8 months of RT in adults with overweight/obesity, although energy intake relative to fat‐free mass was reduced. This longer intervention may be more comparable to the present study, where no overall differences in body weight‐normalized energy intake were observed across the intervention period, despite evidence of increased energy intake during rest days and following RTET. Collectively, human studies do not consistently show that chronic RT suppresses or increases energy intake, and interpretation is limited by self‐reported intake, differences in RT protocols, short intervention durations, and cohorts that are often overweight/obesity‐focused or single‐sex.

From a hormonal perspective, RT produced minimal differences between conditions, with no changes in fasting portal hormone concentrations and no differences in most postexercise appetite hormones, apart from elevated postexercise acyl‐ghrelin in RT conditions. The postexercise findings align with modality‐specific differences in appetite regulation. Acute aerobic exercise has been consistently shown to suppress appetite through reductions in acyl‐ghrelin and increases in GLP‐1, with minimal changes in PYY (McCarthy et al., 2024), whereas RT elicits a more limited hormonal response, typically reducing acyl‐ghrelin without meaningful changes in GLP‐1 or PYY (McCarthy et al., 2024). Acute energy intake responses remain equivocal for both modalities, likely due to variability in timing, intake protocols, and human energy intake assessment (McCarthy et al., 2024). Over chronic interventions, aerobic exercise is more consistently associated with reductions in leptin and variable changes in insulin, alongside inconsistent effects on energy intake (Dorling et al., 2018), whereas RT remains underrepresented, with limited and inconclusive evidence for both hormonal and intake adaptations (Dorling et al., 2018; Panissa et al., 2024). Panissa et al. (2024), who reported decreases in energy intake with chronic RT, found only one study simultaneously examining hormone changes, which showed decreases in acyl‐ghrelin and increases in PYY in men with obesity (Rostamzadeh & Sheikholeslami‐Vatani, 2022). Similarly, Kwon (2025) and Ebal et al. (2007) both reported reductions in body weight and energy intake in male rats following RT, with Ebal et al. also observing decreases in ghrelin and insulin. However, Ouerghi et al. (2021) and Abassi et al. (2025) report that chronic exercise may increase ghrelin, particularly in longer interventions and in the presence of weight loss. Importantly, much of this literature is derived from male‐dominated cohorts (Dorling et al., 2018; McCarthy et al., 2024; Panissa et al., 2024; Rostamzadeh & Sheikholeslami‐Vatani, 2022). While the present findings broadly align with the limited RT literature, the divergence observed between males and females indicates that exercise modality alone does not fully explain energy balance adaptations. Although fasting portal hormones were not altered by RT, males exhibited higher insulin, leptin, and total PYY, whereas females exhibited higher acyl‐ghrelin, indicating a sexually distinct fasting endocrine profile independent of training. The higher acyl‐ghrelin in females and higher leptin/insulin profile in males are broadly consistent with prior rodent evidence of sex‐dependent appetite and metabolic endocrine regulation (Börchers et al., 2022; Fourny et al., 2021; Gómez‐Roncal et al., 2025); however, sex differences in circulating PYY appear less consistent, with prior evidence suggesting that PYY sex differences may be more evident during early postnatal development than in adulthood (Tovar et al., 2004).

Our study demonstrates sex‐specific differences in energy compensation responses to RT, with males exhibiting reduced body weight, while females did not show a comparable reduction despite higher relative training volume. The male‐specific reduction in body weight is consistent with previous RT research in male rats (Ebal et al., 2007; Kwon, 2025), whereas the absence of a comparable effect in females highlights a clear divergence. This divergence is consistent with Foright et al. (2020) demonstrating that chronic aerobic exercise produces sexually dimorphic responses, with males reducing body weight and females increasing energy intake to compensate. We observed that postexercise intake patterns differed by sex, with elevated 48‐h energy intake and increased relative intake in RT groups, and evidence suggesting that these responses were influenced by female‐specific patterns. Similar aerobic findings have been reported by Yang et al. (2020), who showed that females increase energy intake earlier and to a greater extent than males, while males exhibit delayed compensation. Human data support this pattern, with Donnelly et al. (2003) reporting reductions in body weight in males but not females following aerobic exercise. Likewise, Thackray et al. (2016) found that females are more likely to exhibit hormonally mediated increases in appetite in response to the initiation of exercise. Together, these findings support a pattern in which females may exhibit earlier energy compensation, whereas males show greater body weight reductions over comparable intervention periods.

In the present study, RT females exhibited elevated postexercise acyl‐ghrelin, whereas males showed decreased body weight without corresponding increases in ghrelin, indicating a dissociation between endocrine responses and energy compensation outcomes. This is contrary to literature suggesting that increases in ghrelin associated with chronic exercise are attributed to reductions in body weight (Abassi et al., 2025). Because leptin and insulin were unchanged, the divergent energy compensation responses observed here do not appear to be governed solely by traditional tonic appetite regulators (Grgic & Mikulic, 2017; Rubio‐Valles & Ramos‐Jimenez, 2025). Instead, the elevated postexercise ghrelin observed in RT females, but not males, suggests that ghrelin‐mediated compensation may be sex dependent. However, the magnitude of the post‐RTET acyl‐ghrelin response was modest compared with larger preprandial increases reported before entrained meals (Cummings et al., 2001; Drazen et al., 2006). Thus, elevated acyl‐ghrelin should be interpreted as a potential contributor to post‐RTET compensatory feeding rather than as a sufficient causal signal on its own. The aerobic work by Foright et al. (2020), demonstrated reductions in leptin in males but not females and confirmation of similar leptin responsiveness in both, also suggests that long‐term exercise‐induced energy balance outcomes are not directly mediated by classical tonic appetite hormones. The elevated ghrelin in females without accompanying weight loss suggests that factors beyond energy deficit—such as gonadal hormone regulation—may be involved. Börchers et al. (2022) demonstrated that female rats exhibit higher circulating ghrelin levels and increased central sensitivity to ghrelin compared to males, with these differences amplified during fasting, and evidence that ovarian tissue itself may contribute to ghrelin secretion. It is also possible that the expected increase in ghrelin associated with chronic exercise (Abassi et al., 2025) may manifest earlier in females due to more rapid energy compensation, whereas similar responses in males may require longer intervention durations, although this interpretation is not consistent with the body weight outcomes. Collectively, these findings reinforce that energy compensation during exercise is sexually dimorphic and cannot be fully explained by exercise modality or canonical endocrine pathways alone.

Several limitations should be considered. Although the pattern of elevated postexercise acyl‐ghrelin in females supports a role for gonadal regulation of energy compensation, ovarian hormones were not measured; thus, we can only speculate whether hormones across the estrous cycle influence ghrelin. Future studies should directly quantify ovarian hormones and pair these measures with ghrelin and receptor signaling to establish causality. Additionally, females completed a higher relative training volume, introducing a potential confounding effect of training dose. While this reflects consistent sex differences in voluntary and task performance, studies that match RT workload, although not as reflective of individual progressive overload, are needed to isolate sex effects independent of RT stimulus. The intervention duration may have influenced the temporal pattern of energy compensation, particularly given evidence for delayed compensation in males; longer interventions with extended follow‐up would clarify whether male ghrelin and intake responses converge over time. Stress was not assessed. Given that ladder climbing can elicit stress responses that interact with appetite regulation, future work should include stress biomarkers or habituation controls. Body composition was assessed through manual dissection of selected tissues, which introduces potential human variability and limits whole‐body composition assessment, particularly for skeletal muscle. Energy expenditure was also not measured; therefore, we cannot determine whether chronic RT or acute RTET altered total energy expenditure, substrate utilization, or spontaneous activity. Future studies using indirect calorimetry/metabolic cage systems, combined with whole‐body composition assessment, would provide a more complete evaluation of RT‐induced energy compensation. Animals underwent 12 h food deprivation prior to exercise testing. Although this approach standardized pretest nutritional status across groups, food deprivation may have affected outcomes. Therefore, the post‐RTET endocrine and feeding responses should be interpreted as responses to acute RTET under food‐deprived conditions. Although estrous cycle stage was recorded on the day of RTET, the number of animals within each stage was too small to adequately assess whether estrous cycle stage influenced post‐RTET energy intake. Therefore, the post‐RTET estrous cycle‐stage data should be interpreted as descriptive only. Finally, although energy intake and hormones were captured at multiple time points, measurements were not continuous across all recovery periods, which may underestimate the timing and magnitude of compensatory feeding; higher‐resolution intake monitoring including meal size and frequency, and longer postexercise windows would strengthen inference on compensation dynamics.

## 5 CONCLUSION

In summary, this study demonstrates that chronic RT elicits sexually dimorphic energy compensation, with females exhibiting greater compensation than males. This was reflected by decreased body weight in the RT males despite higher relative training volume in females. This divergence is associated with elevated postexercise acyl‐ghrelin in females, while other appetite‐regulating hormones remain unchanged, indicating that RT induces distinct endocrine and behavioral adaptations compared to aerobic exercise. These findings identify ghrelin as a potential mediator of sex‐specific compensation during RT and suggest that energy balance responses to exercise may not be governed by traditional tonic appetite hormones alone. From a translational perspective, these results highlight the importance of considering sex in exercise prescription, particularly when RT is used to modulate body weight and energy intake. This is increasingly important if these findings are replicated in humans, as RT is being suggested to take a more primary role in physical activity guidelines (Chaabene et al., 2025; Phillips et al., 2023). Collectively, this work advances the understanding of exercise‐induced energy compensation by establishing a novel, sex‐dependent framework in which ghrelin acts to regulate energy balance during RT. Moreover, the sexually dimorphic ghrelin actions may be related to gonadal hormones, which future studies will need to confirm.

## AUTHOR CONTRIBUTIONS

**Josh C. Kuzminski:** Conceptualization; data curation; formal analysis; investigation; methodology; project administration; resources; supervision; validation; visualization. **Jared R. Rollingson:** Investigation. **Connor Bunnage:** Investigation. **Jana Katoch:** Investigation. **Marc R. Bomhof:** Conceptualization; funding acquisition; investigation; methodology; project administration; resources; supervision; validation.

## FUNDING INFORMATION

This work was supported by a research grant through the Natural Sciences and Engineering Research Council of Canada (NSERC, RGPIN‐2018‐05091, M.B.).

## CONFLICT OF INTEREST STATEMENT

The authors declare no conflicts of interest.

## ETHICS STATEMENT

All procedures conformed to the guidelines of the Canadian Council on Animal Care and were approved by the University of Lethbridge Animal Welfare Committee (Protocol #2503).

## Data Availability

The data will be made available upon request.
